# First insight into oral health misinformation in Jordan; an analysis of web content and information-seeking behaviors

**DOI:** 10.1371/journal.pone.0325513

**Published:** 2025-06-06

**Authors:** Issam B. Rasheed, Bayan F. Ababneh, Yara I. Al-Habsheh

**Affiliations:** 1 Department of Oral Medicine and Oral Surgery, Faculty of Dentistry, Jordan University of Science and Technology, Irbid, Jordan; 2 Dental Surgery Interns, Faculty of Dentistry, Jordan University of Science and Technology, Irbid, Jordan; Ajman University, UNITED ARAB EMIRATES

## Abstract

**Background:**

Integrating the internet into daily life has profoundly influenced the public's behavior of seeking health information. Free access to cyberspace created a fertile environment for the spread of oral health misinformation, which can have a detrimental impact on the public’s oral health. The prevalence of oral health misinformation in Jordan has not been investigated; therefore, it is crucial to understand how oral health misinformation originates and what contributes to its dissemination.

**Objectives:**

This study aims to examine the prevalence of oral health misinformation published on web pages in Jordan and to offer insight into the public's information-seeking behaviors regarding oral health.

**Methods:**

This study is a mixed methods infodemiological analysis of oral health misinformation. A systematic content analysis was executed on web pages published in the Arabic language in Jordan from 2019 to 2023.

**Results:**

704 web pages were retrieved, of which 320 relevant web pages were included in the content analysis. Among these, 193 web pages (60.3%) published oral health misinformation. Publishers without a professional background published 185 web pages (95.9%) of the total misinformation-expressing web pages. According to the dental field, the highest frequency of misinformation occurred in oral medicine-101 web pages (52.3%). The validity of published oral health information was significantly influenced by the publishers’ interest (P = 0.006), the articles’ main themes (P = 0.005), and the publishers' professional background (P < 0.001). Contextual analysis of oral health misinformation showed significant differences among dental fields (P = 0.019), with the most frequent occurences related to causes (18.8%), home remedies (15.7%), and treatment (15.5%). Geographical variations in interest in oral health searches were observed across Jordanian governorates (P < 0.001), and temporal trends in interest varied significantly across the five-year period(P = 0.019).

**Conclusion:**

The findings of this study suggest a need for public health interventions to restrict the dissemination of oral health misinformation.

## Introduction

The widespread use of the internet has made information-seeking a routine practice in the digital age. Across various demographics, increasing digital literacy has facilitated online searches for health information [1,2]. Social media emerged as a platform for sharing information and experiences related to health. While information technologies have proven effective in promoting health awareness, they have provided a fertile ground where fake news and misleading health information can thrive [3,4]. This spread of information that defies the scientific consensus, or misinformation, became particularly pronounced during the COVID-19 pandemic, which the World Health Organisation described as an “infodemic” [2,5].

The integration of information technology in modern dentistry has become indispensable. From enhancing compliance of patients after dental treatments to promoting oral health awareness, information technology now plays a key role in the public’s oral health [6,7]. On the other hand, social media platforms became free advertising tools that dental professionals use to promote dental services. Its visual appeal and user-friendly nature have encouraged oral health information-seeking. Nonetheless, this pervasive reliance on digital media calls for monitoring and regulation to prevent its misuse [8,9]. Therefore, the widespread availability of information could influence the public’s perception and attitudes toward oral health. Yet, misinformation sourced from unverified web pages contributes to the dissemination of oral health falsehood. When combined with the rapid dissemination power of social media, this potentially creates a self-perpetuating cycle of oral health falsehood.

Enhanced digital literacy and accessibility to information technology have promoted the behaviors of online information-seeking related to dental health. However, it has been shown that shortcomings of such practice include the existence of contradictory and unreliable sources, questionable quality of available information, and the emergence of the ‘internet-prepared patient phenomena' [10,11]. While information-seeking is a key facet of dental care, such barriers might influence the public's oral health attitudes and could impose a detrimental impact on their health if the information is inaccurate [12].

In Jordan, cultural homogeneity facilitates the spread and acceptance of health misinformation. Despite the dialectical disparity between different geographical regions in Jordan, it was shown that social media curtailed such differences and amalgamated the use of a common language [13]. It has been demonstrated that while the consumption of misinformation is driven by selective exposure, the assimilation of health rumors and fake news is affected primarily by the social environment through which information circulates [14]. While user-interactive social media platforms such as Facebook and X allow unrestricted engagement on trending topics, this further facilitates the spread of misinformation, especially while Jordan is among the highest Arab countries in Facebook use [15]. Indeed, the Dynamics of misinformation spread are affected by the platform used [16]. Therefore, the digital social sphere in Jordan calls for careful inspection of sources of health misinformation to contain their dissemination and assimilation by the public. Web pages that contain oral health information is a logical starting point to scrutinize the validity of oral health information.

This study aims to explore the prevalence of oral health misinformation published on web pages in Jordan. Additionally, it seeks to characterize misinformation and to identify underlying factors that influence its spread. Furthermore, the study aims to shed light on information-seeking behaviors of oral health in Jordan.

## Methods

### Study design

This study is a mixed-methods infodemiological analysis designed to explore the landscape of oral health information in Jordan. To achieve this aim, the study seeks to investigate the prevalence of oral health misinformation on web pages and to identify potential constituents that influence the validity of oral health information. Additionally, the study aims to characterize online information-seeking behaviors related to oral health in Jordan. For qualitative analysis, the study seeks to explore themes associated with published oral health information. The target source of information consisted of web pages available in Jordan, published in the Arabic language from 1 January 2019 till 31 December 2023.

### Ethical approval

Content that is published on web pages is considered public information that does not require access authorization. Nonetheless, handling of this information is subject to regulation and legal accountability, according to the Cybercrime Law number 17 of 2023 of Jordan. This study is a content analysis that aims to identify oral health-related misinformation and to characterize factors that influence the validity of oral health information. Therefore, no IRB approval was required to conduct this analysis. However, data obtained from the content analysis was handled carefully. For instance, during content analysis, web page identifiers (URLs) were handled with discretion by the two investigators, and data sets were stored on an encrypted cloud storage specifically designed for this purpose. Furthermore, data sets intended to be shared during the review process were anonymized and cleared of identifying information.

### Data collection

Data collection was conducted using a systematic approach. Webpage screening was performed using the Microsoft Edge browser, with all cookies and browsing history cleared to maintain unbiased results and reduce the impact of personal preferences on search results.

Initially, four search concepts related to oral health in Arabic were selected (Table 1). To ensure inclusive results, “teeth” in Arabic was included in both its common forms, with and without the glottal stop (ء), represented as teeth* and teeth, respectively. This choice reflected common colloquial usage related to the oral cavity in Jordan. The Explore tab in Google Trends was used to retrieve data relevant to the four concepts. The criteria for this purpose were set as follows: the region was set to Jordan, the time range was customized from January 1, 2019, to December 31, 2023, all categories were selected, and a web search was chosen. Each concept run on Google Trends yielded three sets of data: top search queries, interest over time, and interest by sub-region. These datasets were analyzed independently, each serving a distinct purpose within the study. The top search queries were used to retrieve relevant web pages for the purpose of misinformation screening, whereas the interest over time and the interest by sub-region were used to provide an insight into the information-seeking behaviors of web users.

**Table 1:** Concepts related to oral health and their translation to English.

Concept	Translation
الاسنان	teeth
الأسنان	teeth*
الفم	mouth
اللسان	tongue

* teeth with glottal stop depicting (ء) in the Arabic language.

**Fig 1:**
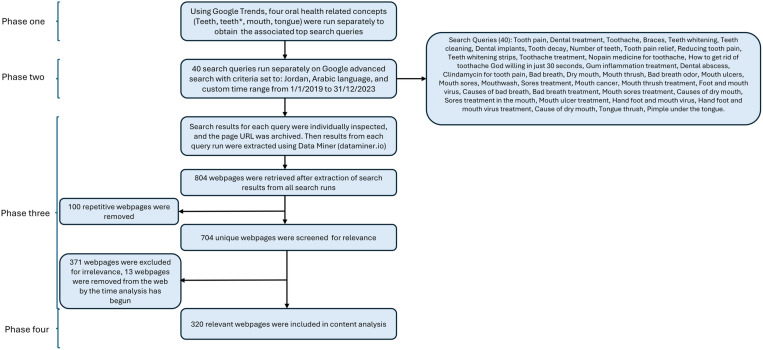
A flowchart illustrating the phases of data collection.

For misinformation screening, the retrieval of web pages relevant to oral health was conducted systematically to ensure careful and inclusive collection of web pages for the content analysis. Fig 1 illustrates the phases of web page retrieval. In phase one, the top search queries obtained from Google Trends were cleaned to remove duplicates and irrelevant items (S1 File).

In phase two, these refined queries were then run using Google Advanced Search, with parameters set as follows: the region was set to Jordan, the language was set to Arabic, and the time range was selected from January 1, 2019, to December 31, 2023. To ensure that search results remained unchanged for screening, retrieved results were archived using Archive Today. Additionally, each search query was assigned an alphabetical code, and the corresponding archived URL was recorded to ensure consistent and systematic screening between investigators. For example, code A was assigned to the first search query “causes of dry mouth” (“أسباب جفاف الفم”), code B to the second search query “causes of bad breath” (“أسباب رائحة الفم”), and similarly for the remaining search queries (S2 File).

In phase three, web pages that resulted from each search query run were then extracted using Data Miner (http://dataminer.io). To ensure inclusive and unbiased search results, two independent and trained investigators (BA, YA) extracted the web pages using the Data Miner tool. Web pages from both examiners were then combined, and duplicates were removed. Additionally, web pages were numbered using the code of the search query, followed by the number of the webpage on the list. For example, web pages from the search query “causes of dry mouth” (“أسباب جفاف الفم”) were numbered as A1, A2, A3, A4, etc., and web pages from the search query “causes of bad breath” (“أسباب رائحة الفم”) were numbered as B1, B2, B3, B4, etc. (S3 File).

### Content analysis

Content analysis comprised the fourth phase of data collection. Between February and August 2024, two blinded investigators (BA and YA) conducted a manual analysis of web pages. Initially, web pages were screened for relevance. Arabic web pages that consisted of articles where the search term was explicitly mentioned in the body of the article were included. For instance, in category A (causes of dry mouth), examiners manually screened the article for the phrase ‘dry mouth’. Web pages that were published in English, that were exclusively marketing or advertisement content, or did not mention the search term in the article were excluded. Results of relevance screening from both investigators were checked for inconsistencies, then combined into one list of included web pages (S4 File).

Manual screening for misinformation in web pages was then conducted. Each web page was categorized as either containing misinformation or not. Detection of misinformation was guided by the criteria established by Swire-Thompson and Lazer. Examples of misinformation include claims that directly defy the scientific consensus or that lack current scientific evidence. For example, the statement ‘drinking fluoridated water causes dental caries’ contradicts the scientific consensus, whereas the claim ‘drug Buspirone causes dry mouth’ lacks an up-to-date supporting evidence.

For this purpose, authoritative sources such as the National Institutes of Health, National Library of Medicine, PubMed Open Access (2015 and later), the American Academy, and Google Scholar (2015 and later) were consulted for validity checks.

Articles from the included web pages were categorized based on three key factors: the publisher’s underlying interest, the article’s main theme, and the publisher’s specialization in the topic. The categorization of publishers’ interest was adapted from the Council of Europe report by Wardle & Derakhshan, and per publishing policies in Jordan. Four interest categories were identified: financial if the interest was promoting a health service, well-being journalism if the article was part of a news webpage, social if the article was published on online blogs dedicated to social interaction within specific social groups, and informational if the article was published by an official authority to promote awareness against deceptive content and beliefs.

For categorization of the article’s main theme, four categories were identified: medical, dental, herbal, or lifestyle. According to the publisher’s specialization, articles were dichotomized as professional or non-professional based on whether writers were specialized or not specialized in the subject matter of the article, respectively. For example, for an article within the category of ‘causes of dry mouth’, if there was no contributor’s note indicating the writer’s health background, the publisher was considered ‘non-professional’. Such a background may be from dentistry, dental hygiene, pharmacy, medicine, or any other health profession that can be considered a reliable source of health information. Web pages published by official health bodies such as the Ministry of Health or the regional UNICEF office in Jordan were considered professional.

For thematic analysis, web pages were classified according to the specific field of dentistry addressed in the article (cariology, general dentistry, endodontics, oral medicine, periodontics, prosthodontics, pedodontics, oral surgery, orthodontics), the outline of the oral health issue being queried by the web user, and finally the context of the oral health issue as described in the content.

For example, web page V3, which was titled *‘immediate treatment for gum inflammation’*, was a journalism article published without an author bio (contributor note) in an electronic newspaper. The article was identified as having oral health misinformation, specifically the claim that peppermint tea treats gingivitis. Based on this, the web page was categorized as follows: the publisher’s interest was classified as well-being journalism, the article’s main theme was dental, the publisher’s background was considered non-professional. The dental specialty addressed was periodontics, the outline of the oral health problem was gum inflammation, and the context of the misinformation was related to treatment and home remedies.

Outcomes of content analysis from both investigators were combined and screened for inconsistencies using a special formula for conditional formatting in Excel. Divergent analyses between the two investigators were revisited and further analyzed until a consensus was reached (S5 File). All misinformation content was listed for each web page (S6 File).

For the analysis of information-seeking behaviors, the interest over time data set was used to demonstrate how popularity of search terms varied across the five years (S7 File). Additionally, the interest by sub-region data set was utilized to indicate how popularity of search terms relevant to oral health varied across the twelve governorates of Jordan between 2019 and 2023 (S8 File). To ensure a more precise reflection of how the interest in search terms varied geographically, popularity was measured across the twelve governorates individually as well as clustered into three geographical regions: Northern region, Midlands, and Southern region.

### Data analysis

Qualitative data analysis was conducted to explore the prevalence of oral health misinformation and factors influencing their spread. To achieve this, the distribution and context of misinformation in six dental specialties were recorded, categorized by publisher interest, article main theme, and publisher background. Fisher’s Exact test was employed to assess the impact of each category on information validity,. Additionally, a contextual analysis was conducted by identifying the nature of oral health misinformation and tracking its distribution across different dental fields. The influence of dental specialty on contextual variation was evaluated using Kruskal-Wallis’s test. Information-seeking behavior was analyzed using the ‘interest over time’ and the ‘interest by sub-region’ data sets. For this purpose, the popularity of the search concepts over time and across the geographical regions was compared separately using Kruskal-Wallis and Dunnett’s multiple comparison tests.

For the qualitative analysis of online content, web pages were screened to identify recurring themes related to oral health information in Jordan. This involved extracting data on dental specialties, the nature of the oral health problem queried by the web user, and the context of any misinformation present. These elements were then illustrated through a conceptual map. All statistical analyses were performed using GraphPad Prism version 10.0.0 for Windows (GraphPad Software, Boston, Massachusetts USA. www.graphpad.com), with the significance level maintained at p < 0.05. The minimal dataset supporting the statistical analyses is provided in the supplementary file (S9 File).

## Results

Among the 320 web pages included for content analysis, 193 (60.3%) web pages were found to have oral health-related misinformation published in their articles. Table 2 summarises the frequency distribution of web pages that contained oral health misinformation. Misinformation-containing web pages were categorized in six identified dental specialties and according to three main criteria: publishers’ interest, the article’s main theme, and publishers’ professional background. The highest frequency of oral health misinformation was found in the field of oral medicine (52.3%), followed by general dentistry (35.2%). Whereas the lowest frequency was found in the fields of orthodontics (1%) and pedodontics (0.5%). According to the publishers’ interest, the majority of oral health misinformation was under well-being journalism (77.2%), whereas social interest had the lowest frequency (2.6%). According to the article’s main theme, the theme with the highest frequency of misinformation was dental articles (54.4%), and the theme with the lowest frequency was herbal articles (10.4%). In the category of Publishers’ background, 95.9% of misinformation-containing articles were published by non-professional publishers.

**Table 2:** Summary of the distribution of misinformation-containing web pages according to the publisher interest (A), article main theme (B), and publisher background (C).

A)		Publisher’s Interest			
	Total	Financial	Informational	Social	Wellbeing Journalism
Cariology	6 (3.1%)	0	0	1 (0.5%)	5 (2.6%)
Teeth decay	6 (3.1%)	0	0	1 (0.5%)	5 (2.6%)
General Dentistry	68 (35.2%)	15 (7.8%)	2 (1.0%)	2 (1.0%)	49 (25.4%)
bad breath	29 (15.0%)	4 (2.1%)	0	0	25 (13.0%)
Braces cleaning	1 (0.5%)	1 (0.5%)	0	0	0
Laser teeth whitening	1 (0.5%)	0	1 (0.5%)	0	0
Mouthwash	12 (6.2%)	3 (1.6%)	1 (0.5%)	0	8 (4.1%)
Teeth cleaning	1 (0.5%)	0	0	0	1 (0.5%)
Teeth pus	1 (0.5%)	1 (0.5%)	0	0	0
teeth whitening	1 (0.5%)	0	0	0	1 (0.5%)
Teeth whitening	8 (4.1%)	0	0	0	8 (4.1%)
toothache	12 (6.2%)	5 (2.6%)	0	2 (1.0%)	5 (2.6%)
Toothache	1 (0.5%)	0	0	0	1 (0.5%)
Yellowish teeth	1 (0.5%)	1 (0.5%)	0	0	0
Oral Medicine	101 (52.3%)	10 (5.2%)	9 (4.7%)	1 (0.5%)	81 (42.0%)
Hand Foot Mouth disease	5 (2.6%)	1 (0.5%)	0	0	4 (2.1%)
Mouth cancer	17 (8.8%)	2 (1.0%)	3 (1.6%)	0	12 (6.2%)
mouth dryness	33 (17.1%)	5 (2.6%)	5 (2.6%)	1 (0.5%)	22 (11.4%)
Mouth sores	34 (17.6%)	1 (0.5%)	1 (0.5%)	0	32 (16.6%)
Mouth thrush	11 (5.7%)	1 (0.5%)	0	0	10 (5.2%)
Tongue thrush	1 (0.5%)	0	0	0	1 (0.5%)
Orthodontics	2 (1.0%)	0	0	0	2 (1.0%)
Braces	1 (0.5%)	0	0	0	1 (0.5%)
Pain from braces	1 (0.5%)	0	0	0	1 (0.5%)
Pedodontics	1 (0.5%)	0	0	0	1 (0.5%)
Teeth decay	1 (0.5%)	0	0	0	1 (0.5%)
Periodontics	15 (7.8%)	3 (1.6%)	0	1 (0.5%)	11 (5.7%)
Gum disease	1 (0.5%)	1 (0.5%)	0	0	0
Gum inflammation	14 (7.3%)	2 (1.0%)	0	1 (0.5%)	11 (5.7%)
Total	193 (100)%	28 (14.5%)	11 (5.7%)	5 (2.6%)	149 (77.2%)
B)		Article Main Theme			
	Total	Dental	Herbal	Lifestyle	Medical
Cariology	6 (3.1%)	2 (1.0%)	3 (1.6%)	0	1 (0.5%)
Teeth decay	6 (3.1%)	2 (1.0%)	3 (1.6%)	0	1 (0.5%)
General Dentistry	68 (35.2%)	38 (19.7%)	5 (2.6%)	24 (12.4%)	1 (0.5%)
bad breath	29 (15.0%)	15 (7.8%)	4 (2.1%)	9 (4.7%)	1 (0.5%)
Braces cleaning	1 (0.5%)	1 (0.5%)	0	0	0
Laser teeth whitening	1 (0.5%)	1 (0.5%)	0	0	0
Mouthwash	12 (6.2%)	9 (4.7%)	0	3 (1.6%)	0
Teeth cleaning	1 (0.5%)	0	0	1 (0.5%)	0
Teeth pus	1 (0.5%)	1 (0.5%)	0	0	0
teeth whitening	1 (0.5%)	1 (0.5%)	0	0	0
Teeth whitening	8 (4.1%)	1 (0.5%)	0	7 (3.6%)	0
toothache	12 (6.2%)	8 (4.1%)	1 (0.5%)	3 (1.6%)	0
Toothache	1 (0.5%)	0	0	1 (0.5%)	0
Yellowish teeth	1 (0.5%)	1 (0.5%)	0	0	0
Oral Medicine	101 (52.3%)	54 (28.0%)	8 (4.1%)	19 (9.8%)	20 (10.4%)
Hand Foot Mouth disease	5 (2.6%)	0	0	0	5 (2.6%)
Mouth cancer	17 (8.8%)	15 (7.8%)	0	1 (0.5%)	1 (0.5%)
mouth dryness	33 (17.1%)	12 (6.2%)	1 (0.5%)	8 (4.1%)	12 (6.2%)
Mouth sores	34 (17.6%)	16 (8.3%)	7 (3.6%)	10 (5.2%)	1 (0.5%)
Mouth thrush	11 (5.7%)	10 (5.2%)	0	0	1 (0.5%)
Tongue thrush	1 (0.5%)	1 (0.5%)	0	0	0
Orthodontics	2 (1.0%)	1 (0.5%)	0	1 (0.5%)	0
Braces	1 (0.5%)	0	0	1 (0.5%)	0
Pain from braces	1 (0.5%)	1 (0.5%)	0	0	0
Pedodontics	1 (0.5%)	1 (0.5%)	0	0	0
Teeth decay	1 (0.5%)	1 (0.5%)	0	0	0
Periodontics	15 (7.8%)	9 (4.7%)	4 (2.1%)	2 (1.0%)	0
Gum disease	1 (0.5%)	1 (0.5%)	0	0	0
Gum inflammation	14 (7.3%)	8 (4.1%)	4 (2.1%)	2 (1.0%)	0
Total	193 (100)%	105 (54.4%)	20 (10.4%)	46 (23.8%)	22 (11.4%)
C)		Publisher Background			
	Total	Nonprofessional			Professional
Cariology	6 (3.1%)	6 (3.1%)			0
Teeth decay	6 (3.1%)	6 (3.1%)			0
General Dentistry	68 (35.2%)	66 (34.2%)			2 (1.0%)
bad breath	29 (15.0%)	29 (15.0%)			0
Braces cleaning	1 (0.5%)	1 (0.5%)			0
Laser teeth whitening	1 (0.5%)	1 (0.5%)			0
Mouthwash	12 (6.2%)	11 (5.7%)			1 (0.5%)
Teeth cleaning	1 (0.5%)	1 (0.5%)			0
Teeth pus	1 (0.5%)	1 (0.5%)			0
teeth whitening	1 (0.5%)	1 (0.5%)			0
Teeth whitening	8 (4.1%)	8 (4.1%)			0
toothache	12 (6.2%)	11 (5.7%)			1 (0.5%)
Toothache	1 (0.5%)	1 (0.5%)			0
Yellowish teeth	1 (0.5%)	1 (0.5%)			0
Oral Medicine	101 (52.3%)	95 (49.2%)			6 (3.1%)
Hand Foot Mouth disease	5 (2.6%)	5 (2.6%)			0
Mouth cancer	17 (8.8%)	14 (7.3%)			3 (1.6%)
mouth dryness	33 (17.1%)	30 (15.5%)			3 (1.6%)
Mouth sores	34 (17.6%)	34 (17.6%)			0
Mouth thrush	11 (5.7%)	11 (5.7%)			0
Tongue thrush	1 (0.5%)	1 (0.5%)			0
Orthodontics	2 (1.0%)	2 (1.0%)			0
Braces	1 (0.5%)	1 (0.5%)			0
Pain from braces	1 (0.5%)	1 (0.5%)			0
Pedodontics	1 (0.5%)	1 (0.5%)			0
Teeth decay	1 (0.5%)	1 (0.5%)			0
Periodontics	15 (7.8%)	15 (7.8%)			0
Gum disease	1 (0.5%)	1 (0.5%)			0
Gum inflammation	14 (7.3%)	14 (7.3%)			0
Total	193 (100)%	185 (95.9%)			8 (4.1%)

To investigate the factors influencing information validity, webpages were categorized based on the validity of information and their occurrence in three categories: publishers’ interest, article’s main theme, and publishers’ professional background (Table 3). According to publishers’ interest, a significant association between interest and information validity was found, showing higher misinformation content in well-being journalism, financial, and socially targeted articles, and lower misinformation content in informational articles (P = 0.006). A similar significant association was found between the main themes of published articles. Dental, herbal, and lifestyle articles had a higher frequency of misinformation compared to valid information, while medically themed articles had a lower frequency of misinformation (P = 0.005). Lastly, the publisher’s background had a significant association with information validity, demonstrating a higher frequency of misinformation published by non-professional publishers (P < 0.001).

**Table 3:** Comparison of information validity among the publisher’s interest, the article’s main theme, and the publisher’s professional background.

Classification→	Publisher’s interest				Article main theme				Publisher’s professional background		
Information Validity↓	Financial	Informational	Social	Wellbeing Journalism	Dental	Herbal	Lifestyle	Medical	Nonprofessional	Professional	Total
Misinformation	28	11	5	149	105	20	46	22	185	8	193
Valid information	21	22	2	82	61	11	21	34	108	19	127
Total	49	33	7	231	166	31	67	56	293	27	320
Fisher’s exact test											
P value	0.006				0.005				<0.001		

**Fig 2:**
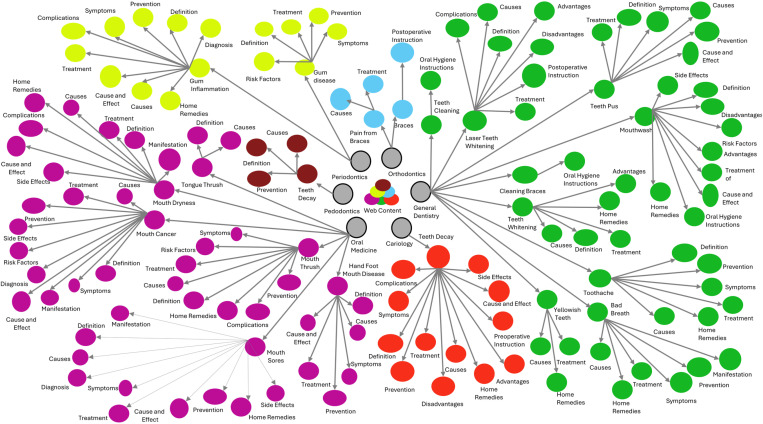
A conceptual map illustrating the distribution of oral health misinformation across dental fields.

Contextual analysis of published oral health misinformation revealed significant variances across the six dental specialties (Table 4). In general, misinformation topics that was most frequently encountered were related to causes (18.8%), home remedies (15.7%), and treatment (15.5%). Different dental specialties demonstrated significant variations in topics (P = 0.019). In cariology, periodontics, and general dentistry, the most frequent topics were found to be related to home remedies, with percentages of 21.4%, 22.4%, and 20.4%, respectively. Whereas within the field of oral medicine, the most encountered topics were related to causes (18.1%) and definition (16.8%). Table 4 shows the contextual distribution of misinformation across the six dental specialties. Fig 2 illustrates a conceptual map of misinformation content.

**Table 4:** Contextual distribution of oral health misinformation among the dental fields.

Context	Total	Cariology	General Dentistry	Oral Medicine	Orthodontics	Pedodontics	Periodontics
advantages	1.5%	7.1%	3.8%	0.0%	0.0%	0.0%	0.0%
cause and effect	3.9%	0.0%	3.8%	3.8%	0.0%	0.0%	6.0%
causes	18.8%	7.1%	21.8%	18.1%	33.3%	33.3%	14.0%
complications	1.3%	7.1%	0.6%	0.8%	0.0%	0.0%	4.0%
definition	12.5%	7.1%	5.8%	16.8%	0.0%	33.3%	14.0%
diagnosis	1.7%	0.0%	0.0%	2.9%	0.0%	0.0%	2.0%
disadvantages	0.6%	7.1%	1.3%	0.0%	0.0%	0.0%	0.0%
home remedies	15.7%	21.4%	22.4%	10.5%	0.0%	0.0%	20.0%
manifestation	4.1%	0.0%	0.6%	7.6%	0.0%	0.0%	0.0%
misuse	0.0%	0.0%	0.0%	0.0%	0.0%	0.0%	0.0%
oral hygiene instructions	1.3%	0.0%	3.8%	0.0%	0.0%	0.0%	0.0%
post-operative instruction	0.6%	0.0%	0.6%	0.4%	33.3%	0.0%	0.0%
pre-operative instruction	0.2%	7.1%	0.0%	0.0%	0.0%	0.0%	0.0%
prevention	8.8%	7.1%	9.0%	8.8%	0.0%	33.3%	8.0%
risk factors	2.6%	0.0%	0.6%	4.2%	0.0%	0.0%	2.0%
side effects	2.6%	7.1%	1.3%	3.8%	0.0%	0.0%	0.0%
symptoms	8.2%	7.1%	2.6%	11.3%	0.0%	0.0%	12.0%
treatment	15.5%	14.3%	21.8%	10.9%	33.3%	0.0%	18.0%
Total	100.0%	100.0%	100.0%	100.0%	100.0%	100.0%	100.0%
Kruskal-Wallis statistic	13.57						
P value	0.019						

Analysis of search behaviors was performed using the ‘interest over time’ and the ‘interest by sub-region’ data sets. Table 5 summarizes the popularity distribution of search concepts across the five years. Analysis of temporal trends revealed variances in the use of search concepts across the five years. Interest in ‘tongue’ remained stable during the timeframe. However, interest in the search concept ‘mouth’ increased remarkably from 2019 to 2023. Overall, a significant variation in the frequency of search concepts from 2019 to 2023 was noted (P = 0.019). A significant difference was found between the terms ‘teeth’ and ‘tongue’, reflecting varied interest in oral health queries (P = 0.041). Geographically, analysis of interest in the four concepts across the twelve governorates of Jordan showed significant differences, indicating variations in search queries related to oral health in different regions (P < 0.001) (Table 6). Furthermore, clustering governorates into three geographical areas similarly indicated the presence of significant differences in the popularity of oral-health related queries (P = 0.017) (Table 7).

**Table 5:** The popularity of oral health-related search concepts across five years in Jordan, according to Google Trends.

Year/Search Concept	Teeth	Teeth*	Mouth	Tongue
2019	73	27	62	50
2020	65	49	61	49
2021	63	57	64	52
2022	70	68	74	54
2023	70	67	81	52
Kruskal-Wallis statistic	9.968			
P value	0.019			
Dunn’s multiple comparisons test	Mean rank diff.	Adjusted P Value		
Teeth vs. Teeth*	6.9	0.389191		
Teeth vs. Mouth	1	>0.999999		
Teeth vs. Tongue	10.1	0.041306		
Teeth* vs. Mouth	−5.9	0.686536		
Teeth* vs. Tongue	3.2	>0.999999		
Mouth vs. Tongue	9.1	0.089394		

*teeth with glottal stop depicting (ء) in the Arabic language.

**Table 6:** The distribution of oral health-related search concepts across the twelve governorates in Jordan.

Search Concept/ Governate	Ajloun	Amman	Aqaba	Balqa	Irbid	Jerash	Karak	Ma’an	Madaba	Mafraq	Tafilah	Zarqa
teeth	91	76	58	77	100	81	78	82	79	90	85	98
teeth*	93	65	61	62	75	95	87	84	82	82	100	74
mouth	100	72	60	71	86	91	89	80	85	80	90	84
tongue	100	65	60	67	93	87	96	83	89	88	96	79
Kruskal-Wallis statistic	33.28											
P value	<0.001											

*teeth with glottal stop depicting (ء) in the Arabic language.

**Table 7:** The distribution of oral-health-related search concepts across the three geographical regions in Jordan.

Concept/Region	Northern	Midlands	Southern
teeth	91	83	76
teeth^*^	86	71	83
mouth	89	78	80
tongue	92	75	84
Kruskal-Wallis statistic	8.19		
P value	0.017		

* teeth with glottal stop depicting (ء) in Arabic.

## Discussion

To our knowledge, this is the first study to address the issue of oral health misinformation published in the Arabic language. Arabic, spoken by four hundred million speakers in twenty-two countries, is also the official language of Jordan. The findings of this study confirm that oral health misinformation is prevalent on web pages sourced from Jordan, suggesting a significant public health concern. The validity of oral health information was influenced by publishers’ professional backgrounds, web pages’ interest in publishing oral health topics, and articles’ themes. Additionally, the behaviors of online information-seeking about oral health varied across different regions and over time in Jordan. The findings of this study provide valuable insights into the evolving landscape of oral health information in Jordan.

These findings underpin the key role of professional health institutions such as universities and health authorities in educating the public on health-related issues. This study showed that informational websites, primarily affiliated with official health and educational bodies, were associated with a higher prevalence of valid oral health information. In contrast, non-professional publishers significantly contributed to oral health misinformation. This reinforces the inconsistent reliability of these sources for oral health information. Wei et al. implied that the inclusion of universities in decision-making and informational input on public health crises is mandatory for enhancing public awareness in challenging times [17]. Similarly, a systematic review by Tursonbayeva et al. suggested a limited governmental role in integrating the public’s input on health-related issues using information technology [18]. Collectively, these findings underscore the informative role of health authorities in preventing health information falsehood by establishing communication channels with the public and implementing efforts of academic institutions toward digital health literacy [19].

In addition, the outcomes of this analysis demonstrate how information platforms interfere with the phenomenon of health misinformation. Journalism is a powerful tool that can influence the public’s behavior. Publishing dentistry-related articles on topics such as home remedies and educational materials increased during the COVID-19 pandemic [20]. Indeed, this study showed that the largest collection of oral health articles (231 out of 320) was published as part of well-being journalism. In Jordan, a study revealed that online news web pages are trusted due to diverse coverage and lower restrictions, which makes the news reported more credible to the public [21,22]. Nonetheless, this study showed that the highest frequency of oral health misinformation also occurred in well-being journalism. This can easily aggravate the adversity of oral health falsehood when combined with the pervasive use of social media platforms such as Facebook, the most widely used social application in Jordan [15,23].

The public’s awareness of oral health issues plays a key role in shaping the behavior of seeking dental and oral care. Investigating awareness among the public and professionals of oral potentially malignant lesions has been a focus in recent years in efforts to promote early detection and prevention of oral cancer [24,25]. Several studies have indicated limited awareness of oral lesions and reliance on practices that hinder early detection, such as the use of home remedies [26,27]. This is concordant with findings from this study, which indicate that oral medicine had the highest collection of articles on different topics and showed the highest frequency of misinformation. Similarly, topics relevant to general dentistry demonstrated the second-highest frequency of misinformation. Implications from these observations are twofold. First, the low awareness of oral health issues about soft tissue and non-dental diseases of the mouth leads to seeking information from online sources. Such predicaments can be mitigated by increasing the attention of professionals to non-dental ailments of the oral cavity and improving the public’s awareness of such ailments. Second, barriers to accessingprofessional dental care contribute to the public’s dependance on online sources for alternative solutions. Understanding such barriers among various social groups is mandatory for improving the public’s oral health [28].

Understanding the public’s behavior of information-seeking could offer insights into preventive measures to control the circulation of health misinformation. Zimmerman et al. argued that health information-seeking is a complex practice that involves both cognitive and behavioral aspects of individuals, which dictate the adoption of the concept of health information-seeking behavior [29]. This study offered multifaceted insights into the public’s practices for obtaining information related to oral and dental issues. Notably, key areas of interest included the causes of oral diseases, home remedies, and treatment options. These patterns offer multiple standpoints. The public’s curiosity about the causes of disease may reflect health anxiety, hypochondriasis, or other psychological factors that influence decision-making and treatment preferences. MacMullan et al. suggested that health anxiety could be one of several factors that drive health information-seeking, which in turn could be a counteracting behavior influencing health decisions [30]. On the other hand, home remedies are used for the treatment of a myriad of ailments by people from varying ethnic and social backgrounds [31,32]. During the COVID-19 pandemic, online searches for home remedies to manage oral health issues have increased remarkably, indicating the rising need for alternative solutions during periods of restricted access to dental care [33,34]. However, other factors may explain this rising information-seeking trend in Jordan. This study showed that online seeking behaviors for oral health information could reflect a deeper problem of the public’s attitudes toward and perception of dental and oral health.

In Jordan, access to dental care is influenced by several factors, most prominently the public’s socioeconomic background and the cost of dental services [35,36]. These barriers have contributed to a higher prevalence of dental problems, especially in underprivileged communities such as those in southern governorates [37]. Such observations are consistent with the findings of this study. Interest in oral health-related concepts varied significantly across the twelve governorates. Additionally, when governorates were grouped into three geographical regions,significant regional differences in interest were also observed. For instance, the popularity of related search concepts was the highest in Ajloun and Tafilah governorates and the lowest in Amman, Aqaba, and Balqa governorates. This is due to the metropolitan differences that exist between southern and midland governorates, which include the capital Amman. According to the Department of Statistics in Jordan, nearly 8% and 29% of the population live in southern and northern governorates, respectively [38]. Moreover, the items of the highest interest varied between regions. For instance, the highest popularity in the northern region was for ‘tongue’, whereas it was for ‘teeth’ in the southern region. This could imply that soft tissue lesions are more prevalent in northern regions compared to southern regions, or that general practice dentistry in the North is more accessible due to a higher population count compared to the southern regions, making people search for basic problems more in the South or higher awareness of soft tissue parts in the north. These findings suggest that disparities in dental care accessibility influence the publics' behaviour in seeking oral health information online. 

The COVID-19 pandemic impacted the use of information technology worldwide. The use of social media platforms and the web to search for oral health-related information has risen significantly during and after lockdown. This is due to limited or nonexistent access to dental services. This surge in health-related content created an infodemic wave that fueled health anxiety. Oral health was among the several health aspects that COVID-19 has remarkably impacted. In Jordan, despite the lack of studies examining clinical parameters of oral health in the post-COVID-19 period, it has been shown that dental care was significantly diminished due to lockdown measures and public fears of health hazards linked to COVID-19 [39]. This has caused further sequels on mental well-being, which may helpexplain the rise of information-seeking behaviors online in compensation for their lack from professional sources [40]. This study has shown significant differences in search terms’ popularity across the five-year time period. However, few search terms showed variations in interest throughout the selected years. For instance, the popularity of the terms ‘mouth’ and ‘teeth’ demonstrated a steady increase in popularity throughout the five years. This could be attributed to increased awareness of oral hygiene, potentially driven by the economic impact of COVID-19. It may also reflectsocial changes such as increased time spent at homedue to unemployment or the shift to remote work. These observation are consistent with a previous report suggesting deterioration of oral health among the public following the COVID-19 pandemic [41].

Addressing the impact of misinformation requires multifaceted approaches. Swire and Lazer demonstrated that tackling the effects of misinformation can be achieved through diverse efforts. These include collaboration between information seekers and physicians, web tools to inspect reliability of sources, enhancing digital health literacy, source quality checks, and encouraging scientists to be more involved in social media [5]. Such approaches provide promising outcomes; however, their effectiveness requires earnest efforts and varies depending on the target population. For instance, the success of enhancing digital health literacy in the elderly population is influenced by several factors such as the individuals' health, the cultural and social barriers, and digital literacy [42,43]. On the other hand, several Western countries have adopted the concept of fact-checking to monitor online information and debunk myths and fake news [44]. In Arab countries, several models have been proposed as reliable tools for fact-checking of public information [45,46]. Nonetheless, these models work by textual and linguistic analysis rather than fact-checking. Health information requires evidence-based checks as it constantly evolves.

While this study offers valuable insight into the landscape of oral health information in Jordan, its findings should be interpreted with caution due to potential limitations. The information-seeking behaviors observed do not necessarily reflect the public’s true interest in oral health. Data sourced from Google Trends represents relative search interests in oral health topics rather than absolute search volumes. Therefore, it is difficult to draw definitive conclusions about how interest in the relevant topics varies geographically and over time. Future work could leverage advanced AI-driven tools that account for population size, yielding more precise adjustments and a deeper understanding of how social, cultural, and geographical factors influence this behavior.

Another potential caveat of this study is the lack of social media analysis for oral health misinformation. While the findings of this study showed that web pages serve as a primary source of oral health information, social media platforms facilitate the dissemination of oral health information rapidly and promote the assimilation of oral health misconceptions [4,47]. Future work is needed to explore how tools and user interfaces of different social media platforms influence the assimilation of health information, and to implement social media in promoting health awareness and digital health literacy.

Finally, this study focused on content exclusively retrieved from web pages in Jordan. However, online searches for oral health information may also yield web pages from other Arabic-speaking countries, potentially influencing the information landscape. Such overlap may contribute to an added pernicious effect of health misinformation. On the other hand, a few observations in neighbouring countries prompted similar unguided interactions with online health sources. For instance, a study in Syria found that young adults consider Facebook a trusted source of dental information [48]. In contrast, while Twitter is the most widely used social application in Saudi Arabia, Al-Khalifa et al. implied that it is among the least utilised for promoting oral health awareness [49]. Together, these observations indicate the diverse roles of social media platforms in shaping the landscape of oral health across different regions in the Middle East.

Despite the linguistic and cultural unity shared by neighbouring countries in the Middle East and the Gulf region, substantial differences exist in their political and constitutional frameworks. These disparities influence media and publishing practices. Notably, media and publications in the Middle East operate in a culturally and politically sensitive environment [50,51]. For instance, an alcohol-containing mouthwash may be disregarded by readers despite proven efficacy in combating a certain type of oral disease. Understanding such sociopolitical influences could provide insight into how online health education is received, perceived, and shared across these regions. Additionally, a broader analysis is necessary to assess the need for a validity-check tool that could offer comprehensive coverage in the Arabic region. Platforms that offer validity checks for Arabic content may exist; however, scientific content requires a specialized tool as it evolves constantly.

While limitations existed in this study, the strengths of this work were the vast and varied pool of oral health-related search strings. This ensured the inclusion of all search results that contain the search concept. For instance, instead of building up dental-related queries based on professional knowledge (e.g., dental caries), search strategies were obtained based on the search trends and colloquial phrases of public readers (tooth decay, toothache, tooth pain). In addition, two blinded examiners reviewed and analyzed the published contents. Furthermore, this analysis is the first to underpin the interests and motifs of publishing oral and dental information in the Middle East region.

In conclusion, online oral health misinformation was influenced by publishers’ professional realm and interest in publishing health-related topics. While this does not necessarily indicate a causal relationship, it offers insight into an equivocal aspect of digital health publication in Jordan. Validity checks are essential to maintain a healthy environment of information. A wider and more in-depth behavioral analysis is needed in the future to decipher the public’s attitudes and perceptions of online oral health information. This, alongside the engagement of dental professionals, is crucial to investigate how this prior information exposure is affecting dental care.

## Supporting information

S1 File Top search queries related to oral health. The top search queries represent the most popular search terms relevant to oral health that has been used in the specified time period. 40 search terms (strategies) were obtained from Google Trends.
(XLSX)

S2 File Search run results. The result pages of search runs coded and archived on Archive Today.
(XLSX)

S3 File Web pages extracted from all search runs. List of all web pages extracted from all search runs. 704 web pages were obtained.
(XLSX)

S4 File Relevant web pages. List of web pages obtained after relevance screening (333 web pages).
(XLSX)

S5 File Content analysis spreadsheet. Categorization of web pages and misinformation according to publisher’s interest, article’s main theme, publisher’s specialization, field of dentistry, oral health problem, and context of misinformation.
(XLSX)

S6 File List of misinformation. List of Content of oral health-related misinformation for each web page.
(XLSX)

S7 File Interest over time data set. List of search terms popularity from 2019 to 2023.
(XLSX)

S8 File Interest by sub-region data set. List of search terms popularity across the twelve governorates of Jordan.
(XLSX)

S9 File Minimal dataset. All minimal data used for the statistical analyses, organised according to the table number.
(XLSX)
